# Care-Seeking Behavior of Families of North Indian Children Suffering From WHO-Defined Severe Community-Acquired Pneumonia: A Hospital-Based Prospective Study

**DOI:** 10.7759/cureus.41953

**Published:** 2023-07-16

**Authors:** Krishna Yadav, Shally Awasthi

**Affiliations:** 1 Department of Pediatrics, Dr. Ram Manohar Lohia Institute of Medical Sciences, Lucknow, IND; 2 Department of Pediatrics, King George's Medical University, Lucknow, IND

**Keywords:** under-five mortality, under-five children, care-seeking behavior, community-acquired pneumonia, delayed care

## Abstract

Background

Community-acquired pneumonia (CAP) is one of the leading causes of death in children under five. In developing countries, delayed treatment seeking has been associated with mortality and morbidity. There are only a few studies in India evaluating care-seeking behavior, particularly in children with CAP.

Methods

The present study was a hospital-based prospective semi-qualitative study. The study was conducted on parents or caregivers of consecutively hospitalized children under five (two to 59 months) with WHO-defined severe CAP along with radiological abnormalities consistent with CAP. Categorization of CAP and interpretation of chest X-rays (CXR) were done as per WHO criteria. Complicated CAP was categorized as severe pneumonia and had additional characteristics, including pleural effusion/empyema/pneumothorax requiring intercostal drainage, acute respiratory distress syndrome, or septic shock.

Results

After the screening of 420 consecutively hospitalized children under five with WHO-defined severe CAP along with radiological abnormalities consistent with it, 350 children were recruited in the present study. Among the recruited children, 58.6% experienced delayed care seeking, and among delayed care seekers, 94.6% presented with complications or developed complications during their hospital stay. The median delay in medical attention was three days. It also found that mothers with education levels below a high school had delayed care-seeking behavior. Mothers noticed the illness first in the majority of subjects (190, 54.3%), followed by fathers (78, 22.3%). Visiting traditional healers (46, 22.4%) and opting for home-bound remedies (44, 21.5%) were among the most common reasons for delayed care seeking. Fast breathing was the most concerning symptom among the parents and caregivers of the hospitalized children due to severe CAP followed by retractions, cough, and drowsiness. Retractions, drowsiness, and inability to feed were significantly recognized as alarming symptoms by the parents and caregivers in children with complicated CAP. Delayed care-seeking behavior was more prevalent in families from rural areas than in urban areas. If decision takers were in close relation with the sick child, chances of delayed care were less and vice versa. In urban areas, mothers can make decisions in significantly higher numbers than in rural areas, while grandmothers were more involved in decision-making in rural areas.

Conclusion

The delayed care-seeking behavior was significantly higher in children with complicated CAP. Delayed care-seeking behavior was more prevalent in families from rural areas than in urban areas. The most common reasons for delayed care-seeking behavior were home remedies and visiting traditional healers. Caregivers need to be more aware of the danger signs of CAP and the consequences of treatment delay.

## Introduction

Community-acquired pneumonia (CAP) is one of the main causes of death in children under the age of five, particularly in developing nations [1]. CAP contributes to 15% of all under-five deaths globally [1]. Around 95% of these deaths take place in underdeveloped nations [2]. Children and families worldwide are impacted by CAP, but South Asia and sub-Saharan Africa are where it is most widespread [1]. India alone contributes 20% of global under-five deaths due to CAP [3]. The economic burden of management and prevention is enormous for developing nations. United Nations Organisation (UNO) adopted the Sustainable Development Goals (SDGs) to end the preventable deaths of under-five children by 2030, along with other goals [4]. In underdeveloped nations, delayed treatment-seeking has been linked up to 70% of child deaths, including those from CAP [5]. It has been noted that high death rates have been mainly due to delayed care seeking for a variety of reasons, including ignorance of danger signs, in some areas despite adequate availability of healthcare facilities [5]. There are not many studies examining care-seeking behavior in Indian children, especially those with CAP [6]. Among the available data, there is huge variability in care-seeking behavior and access to healthcare facilities in different communities at the national and regional levels, even within the healthcare system, notably for common and serious illnesses in children [7,8]. Few studies have suggested that care-seeking behaviors are affected by various factors, including affordability, availability of transport facilities, distance, maternal education, socioeconomic status, and health-related cultural practices and preferences [9,10].

Therefore, understanding mothers' and their families' approach toward CAP is important for policy-making to decrease mortality related to CAP in children. Policies addressing health care infrastructure and manpower may not be effective in decreasing mortality because of poor addressing of the care-seeking behavior of needy people of a particular region. The objective of the present study was to understand the social obstacles that prevent children with severe community-acquired pneumonia from timely receiving care seeking.

## Materials and methods

The present study was a hospital-based prospective semi-qualitative study. This was conducted in a tertiary care referral and teaching hospital situated in northern India, namely King George's Medical University Lucknow. The study was conducted on parents or caregivers of consecutively hospitalized children under five (two to 59 months) from July 2015 to June 2017 in the department of pediatrics with WHO-defined severe CAP along with radiological abnormalities consistent with CAP. The children with suspected or proven tuberculosis, cystic fibrosis, bronchial asthma, congenital heart disease, and malignancy or immunocompromised were excluded from the study. Consent from either parents or legal guardian was taken prior to recruitment to the study. Institutional ethical committee has approved the study (Ref.444/R-Cell/65thECM-IIC/P14).

In general, 30 participants are considered to be sufficient for a qualitative study. The sample size for the current study, which was a subset of another study, was taken to 350. Categorization of CAP and interpretation of chest X-rays (CXR) were done as per WHO criteria [11,12]. The delayed care-seeking behavior was considered if parents or caregivers did not visit a qualified health care provider (i.e., registered medical practitioner, RMP) within 24 hours of the onset of symptoms. One-day delayed medical attention was considered when parents or caregivers paid a visit after 24 hours of the onset of symptom(s) but before 48 hours. Complicated CAP was defined as severe pneumonia with additional features such as acute respiratory distress syndrome, septic shock, or pleural effusion/empyema/pneumothorax requiring intercostal drainage. The standard criteria were used for defining these complications [13,14]. 

Data was collected through paper-based questionnaires. These consisted of closed-ended questions with multiple choices. Only one response was asked to choose. The questions and their choices were pronounced by one of the authors in understandable language if the caregivers were unable to read or understand. Only the mother would have been chosen when both parents were available. The preference order of caregiver for responses was mother followed by father, grandmother, grandfather, and then others. Clinical data were extracted from case records prospectively. Data were entered into SPSS version 23 (IMB Inc., Armonk, New York) for statistical analysis. Analysis done was mostly descriptive, and crude OR were estimated along with two-tailed p-value by Chi-squared test where proportions were in case-control status. A p-value less than <0.05 was considered statistically significant. 

## Results

**Figure 1:**
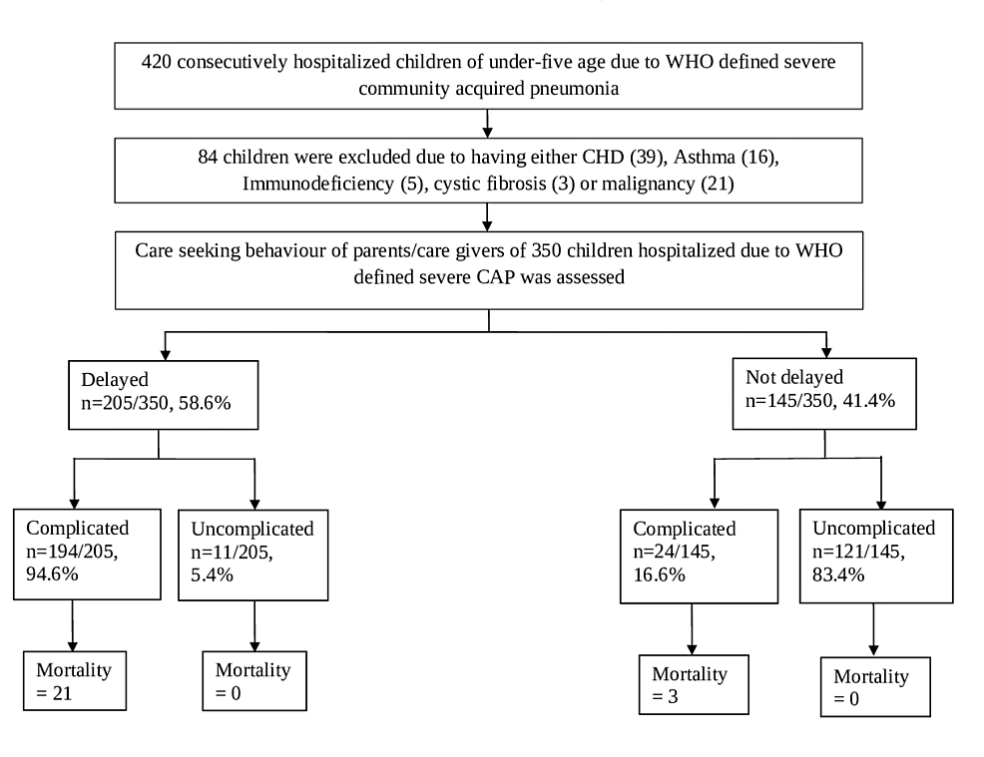
Flowchart of the study

After the screening of 420 consecutively hospitalized children under five with WHO-defined severe and radiological abnormalities consistent with CAP, 350 children were recruited in the present study. The mean ± SD of the age of recruited children was 22.79 ± 20.24 months, and 64.6% were boys. The most common respondents were mothers (205, 58.57%), followed by fathers (80, 22.86%), grandmothers (32, 9.14%), grandfathers (13, 3.72%), and others (20, 5.71%). Among the recruited children, 58.6% were subjected to delayed care-seeking, and among delayed care seekers, 94.6% presented with complications or developed complications during their hospital stay. Mortality was observed in 24 (6.8%) among 350 recruited children in the present study. All these had complicated CAP. There was no mortality among uncomplicated CAP. Mortality was significantly higher among the delayed care-seeking group (21, 10.2%) in comparison to the non-delayed group (3, 2.1%) (p=0.003) (Figure 1, Table 1).

**Table 1:** Care-seeking behavior of parents/caregivers of hospitalized children under five due to severe community-acquired pneumonia CI - confidence interval, cOD - crude odds ratio, CAP - community-acquired pneumonia ⁎ Chi-square with 1 degree of freedom with Yates correction and two-tailed p-value ** Fisher's exact test ***Unpaired student t-test (t=17.5281, df=348, standard error of difference = 0.605)

	Characteristics	Delayed, N=205 (n, %)	Not delayed, N=145 (n, %)	Total	p-value ^*^ (cOD, 95%CI)
1	Complicated CAP	194, 94.6	24, 16.6	218	<0.001 (88.9, 42-188.0)
	Uncomplicated CAP	11, 5.4	121, 83.4	132	
2	Rural	184, 89.8	111, 76.6	295	0.001 (2.7, 1.5-4.9)
	Urban	21, 10.2	34, 23.4	55	
3	Boys	117, 51.8	109, 48.2	226	<0.001 (2.3, 1.4-3.6)
	Girls	88, 70.9	36, 29.1	124	
4	Nuclear family	27, 13.2	35, 24.1	62	0.008 (2.1, 1.2-3.7)
	Joint family	178, 86.8	110, 75.9	288	
5	Mortality	21, 10.2	3, 2.1	24	0.004** (5.4, 1.6-18.5 )
	Survived	184, 89.8	142, 97.9	326	
6	Duration of hospital stay in days (mean ± SD)	15.59 ± 7.12 (95% CI, 14.65-16.54)	4.98 ± 1.84 (95% CI, 9.61-5.24)	-	<0.001***

The magnitude of delayed medical attention is given in Table 2.

**Table 2:** Magnitude (days) of delayed in care seeking behaviour

Delayed in days	N=205	%	Statics
1	34	16.6	Mean ± SD = 4.26 ± 1.64; median = 3
2	40	19.5	
3	48	23.4	
4	33	16.1	
5	27	13.2	
6	17	8.3	
>7	6	2.9	

The median of delayed medical attention was three days. The family member who noticed the illness first is given in Table 3.

**Table 3:** Family member who noticed the illness first

Family member	N (350)	%
Mother	190	54.3
Father	78	22.3
Grandmother	49	14
Grandfather	12	3.4
Aunt	10	2.9
Uncle	6	1.7
Others	5	1.4

It also found that mothers with education levels below a high school had delayed care-seeking behavior. We found that mothers noticed the illness first in the majority of subjects (190, 54.3%), followed by fathers (78, 22.3%). The symptoms which were recognized by parents or caregivers and considered by them as a serious symptom, and that led to first medical care, is given in Table 4.

**Table 4:** Symptoms considered by parents/ caregivers as serious or dangerous that led to first medical care or advice CAP - community-acquired pneumonia

Symptoms	CAP N= 350		Timely care-seeking N=145 (a)		Delayed care-seeking N=205 (b)		p-value (a vs b)	Uncomplicated CAP (N=132) (c)		Complicated CAP (N=218) (d)		p-value (c vs d)
	n	%	N	%	n	%		n	%	N	%	
Fever ± chills	58	16.6	23	15.9	35	17.1	0.87	21	15.9	37	16.9	0.88
Cough	39	11.1	15	10.3	24	11.7	0.82	13	9.8	26	11.9	0.6
Fast breathing	95	27.1	39	26.9	56	27.3	0.93	37	28	58	26.6	0.8
Retractions	78	22.3	21	14.5	57	27.8	<0.01	19	14.4	59	27.1	<0.01
Drowsiness	30	8.6	6	4.1	24	11.7	0.02	5	3.8	25	11.5	0.02
Vomiting/ recurrent vomiting	12	3.4	5	3.4	7	3.4	0.98	4	3	8	3.7	1
Unable to feed	25	7.1	5	3.4	20	9.7	0.033	4	3	21	9.6	0.03
Cyanosis	6	1.7	0	0	6	2.9	NA	0	0	6	2.8	NA
Chest pain	7	2	0	0	7	3.4	NA	0	0	7	3.2	NA

If there were two or more symptoms recognized together, then according to parents or caregivers, the symptom that was most serious and led to seeking medical attention was taken for consideration for Table 4. Fast breathing was the most concerning symptom among the parents of hospitalized children due to severe CAP, followed by retractions, fever, cough, and drowsiness (Table 4). Retractions, drowsiness, and inability to feed were significantly recognized as alarming symptoms by the parents and caregivers in subjects with complicated CAP (Table 4). There were various reasons for delayed care-seeking in children hospitalized due to severe CAP (Table 5).

**Table 5:** Reason for delayed care seeking by parents/ caregivers

Reasons	N=205	%
Not aware about illness/ thought it a not so serious matter	15	7.3
Decision maker was not present	13	6.3
Lack of money	10	4.9
Lack of transport	17	8.3
Caretaker/ decision-taker had other responsibilities	15	7.3
Season (not favoring seasons like raining, excessive cold or warm)	10	4.9
Birth order/ multiple children	13	6.3
Traditional healer	46	22.4
Home treatment	44	21.5
Long distance	13	6.3

Delayed care-seeking behavior was more prevalent in families from rural areas than in urban areas (Table 1). We found that if decision takers were in close relation with the sick child, chances of delayed care were less and vice versa (Table 6).

**Table 6:** Decision-maker for care-seeking of the north Indian families whose children were hospitalized due to severe community-acquired pneumonia

Decision-maker	Total N=350, %	Not delayed N=145, %	Delayed N=205, %	p-value
Mother	41, 11.7	24, 16.6	17, 8.3	0.03
Father	114, 32.6	45, 31.0	69, 33.7	0.69
Grandmother	70, 20.0	19, 13.1	51, 24.9	0.01
Grandfather	94, 26.9	46, 31.7	48, 23.4	0.11
Aunt	10, 2.9	4, 2.8	6, 2.9	0.93
Uncle	21, 6.0	8, 5.5	13, 6.3	0.93

In urban areas, mothers can make decisions in significantly higher numbers than in rural areas. Similarly, grandmothers were more involved in decision-making in rural areas (Table 7).

**Table 7:** Decision-maker of the north Indian rural vs. urban families whose children were hospitalized due to severe community-acquired pneumonia

Decision-maker	Total N=350, %	Rural N= 295, %	Urban N=55, %	p-value
Mother	41, 11.7	23, 7.8	18, 32.7	0.03
Father	114, 32.6	94, 31.9	20, 36.4	0.69
Grandmother	70, 20.0	63, 21.4	7, 12.7	0.01
Grandfather	94, 26.9	84, 28.5	10, 18.2	0.11
Aunty	10, 2.9	10, 3.4	0, 0	NA
Uncle	21, 6.0	21, 7.1	0, 0	NA
Others	0,0	0,0	0,0	NA

## Discussion

CAP in children often progresses rapidly, resulting in poor outcomes. If caught early, CAP can be treated at home with inexpensive antibiotics. Therefore, a timely visit to healthcare facilities or qualified care-seeking is of utmost importance to decrease morbidity and mortality related to CAP in children under five. Additionally, we observed that children frequently arrive at the tertiary center with late-stage of illnesses such as sepsis, hypoxemia, acute respiratory distress syndrome (ARDS), respiratory failure, and empyema of CAP. This could be because of several reasons like unavailability of health centers near their homes, long distances without transport facilities, delayed referral, or delayed care-seeking behavior. Therefore, knowledge of social and economic barriers and factors affecting care seeking behaviors of the community will help to design a health program that will be more comprehensive and useful to the underprivileged community. 

In the present study, authors tried to understand the social barriers responsible for the delayed care-seeking behavior of caregivers without addressing which it is difficult to achieve the SDG targets. Authors found delayed care-seeking behavior was more prevalent in rural areas, living in joint families, and if sufferers were girls. Delayed care-seeking behavior was also significantly associated with complications. It also found that mothers with education levels below a high school had delayed care-seeking behavior. There was a lack of knowledge of danger signs and a lack of power to make decisions mothers were significantly associated with delayed care-seeking behavior. 

The study site being a government tertiary care center, subjects were only severe cases of CAP as defined by WHO because hospitalization of children was mostly for the severe category only. 

In agreement with our results, there are studies that reported similar findings. A study done by Awasthi et al. [15] showed in Indian states (Uttar Pradesh and Bihar), fast breathing was not recognized as an early sign of CAP by caregivers. However, chest indrawing (retraction) was recognized and considered as a danger sign. Most mothers consulted non-qualified local practitioners. In the current study, authors observed that living in a nuclear family was associated with less delayed care-seeking behavior than living in a joint family. In urban areas, mothers were able to make decisions, while in rural areas grandfathers were making decisions. Although there was no difference in resultant delayed and not delayed care-taking behavior when grandfathers were decision-makers, but if mothers were decision-makers, then there was significantly less delayed care-seeking behavior. If the decision had to be made by grandmothers, there was a significant delay in care-seeking. This result is probably because of the ease of decision-making in a nuclear family, while it is difficult in a joint family due to the complex responsibilities and social interactions of various family members.

May et al. [16] reported in their community-based qualitative study that there was a huge trust in quacks (non-registered medical practitioners) in northern India, resulting in delayed qualified medical care. Home-based remedies and visiting traditional healers were the most prevalent reason for delayed care-seeking in the present study. Several other studies especially done in South Asian countries, reported the prevalence of home remedies, self-medications, and frequent visiting of traditional healers resulting in delayed qualified medical care [6, 17-19].

The median delay in care-seeking behavior is usually two to three days in developing countries. Although, early and rational use of antibiotics in bacterial pneumonia is crucial for the prevention of the development of complications and mortality. In the present study, the median delay was for three days, while in other studies, it was two days [5,14].

In the present study, caregivers considered retractions, drowsiness, and inability to feed as serious conditions. There are several studies [6,14] that reported that not only caregivers but first contact service providers, including traditional healers (quacks), accredited social health activists (ASHA), auxiliary nurses, and midwives (ANM), were not able to recognize these signs early and resulting delayed qualified medical care and referral. Therefore, this is a need for hours to train our rural first-contact health professionals to be well-versed with these signs and referral systems. 

In the present study, delayed care seeking was more prevalent when sufferers were girls in comparison to boys. In Indian settings, especially in rural areas, this is a common problem [20]. Without educating mothers and making them able to earn (financially secure), it will be difficult to prevent gender biases in care-seeking behavior in developing countries. 

The present study also had certain limitations. It would be better if a community-based study was done. We could not comment on the health care-seeking behaviors of those who visited private-sector healthcare facilities. 

## Conclusions

The delayed care-seeking behavior was significantly higher in children with complicated CAP. Delayed care-seeking behavior was more prevalent in families from rural areas than in urban areas. The most common reasons for delayed care-seeking behavior were home remedies and visiting traditional healers. The caregivers are needed to be more aware of the danger signs of CAP and the consequences of treatment delay. Home-based remedies and visiting traditional healers are still big hurdles in developing countries like India. Mothers' education and awareness about danger signs are very important to change care-seeking behavior, along with the availability of qualified medical centers and transport. 
